# Case report: Preemptive intervention for an infant with early signs of autism spectrum disorder during the first year of life

**DOI:** 10.3389/fpsyt.2023.1105253

**Published:** 2023-05-03

**Authors:** Costanza Colombi, Natasha Chericoni, Stefania Bargagna, Valeria Costanzo, Raffaella Devescovi, Flavia Lecciso, Caterina Pierotti, Margherita Prosperi, Annarita Contaldo

**Affiliations:** ^1^Stella Maris Foundation (IRCCS), Calambrone, Italy; ^2^Institute for Maternal and Child Health Burlo Garofolo (IRCCS), Trieste, Friuli-Venezia Giulia, Italy; ^3^Department of History, Society and Human Studies, University of Salento, Lecce, Apulia, Italy; ^4^UFSMIA Valdera-Alta Val di Cecina, Azienda USL Toscana Nord Ovest, Pisa, Tuscany, Italy

**Keywords:** preemptive intervention, parent mediated intervention, ESDM, autism spectrum disorder, early development

## Abstract

Autism spectrum disorder (ASD) includes neurodevelopmental conditions traditionally considered to bring life long disabilities, severely impacting individuals and their families. Very early identification and intervention during the very first phases of life have shown to significantly diminish symptom severity and disability, and improve developmental trajectories. Here we report the case of a young child showing early behavioral signs of ASD during the first months of life, including diminished eye contact, reduced social reciprocity, repetitive movements. The child received a pre-emptive parent mediated intervention based on the Infant Start, an adaptation of the Early Start Denver Model (ESDM), specifically developed for children with ASD signs during the first year of life. The child here described received intervention from 6 to 32 months of age, in combination with educational services. Diagnostic evaluations performed at several time points (8, 14, 19, and 32 months) showed progressive improvements in his developmental level and ASD symptoms. Our case study supports the possibility of identifying ASD symptoms and providing services as soon as concerns emerge even during the first year of life. Our report, in combination with recent infant identification and intervention studies, suggests the need for very early screening and preemptive intervention to promote optimal outcomes.

## Introduction

Autism spectrum disorder (ASD) includes heterogeneous conditions characterized by immaturities and atypicalities in social communication and by the presence of restricted patterns of behaviors and interests (1). Research on early development suggests that brain and behavioral atypicalities emerge during the first year of life in children later diagnosed with ASD (2, 3). For example, in their study of infants at high and low familial risk for ASD, Jones and Klin (2) showed a decline in eye contact, as measured through eye-tracking technology, between 2 to 6 months of age in infants later diagnosed with ASD. Moreover, Bosl et al. (3) showed that in infant siblings a diagnosis of ASD could be predicted with high accuracy using EEG measurements as early as 3 months of age.

Importantly, research shows that interventions delivered during the first 2 years of life lead to greater impact on developmental trajectories and symptom severity in comparison to interventions started later (4). In Lombardo et al.’s study (4), children starting intervention during the first 24 months of age demonstrated predictable gains, while it was not the case for children starting intervention later. Moreover, in their recent study, Guthrie et al. (5) showed that children starting a parent-mediated intervention, the Early Social Interaction (ESI) model, at 17 months of age, showed greater gains in receptive/expressive language, social communication, and daily living skills in comparison to children beginning the same intervention at 27 months of age. In order to increase access to intervention as early as possible for young children at high likelihood of receiving a diagnosis of ASD, researchers have developed interventions that can be delivered by teaching strategies to parents. Brian et al. (6), in their large community implemented study, showed that an evidenced-based parent-mediated intervention, the Social ABCs, for toddlers with ASD (age range: 14–34 months) can be delivered within community services. Indeed, parents learned the intervention at fidelity level and toddlers made clinically meaningful gains, suggesting that this approach is feasible and effective and may be proposed to families immediately in response to first signs of ASD. Furthermore, it seems plausible that very early parent-mediated intervention may set children on an accelerated learning trajectory, resulting in fewer resource needs later in development (7).

Research on intervention within the first year of life is very limited, so far only a handful of studies have evaluated the feasibility and the efficacy of parent-mediated intervention for symptomatic infants before 15 months of age. Both Rogers et al. (8) and Whitehouse et al. (9), using, respectively, an adapted version of the Early Start Denver Model [ESDM; Rogers et al. (10)], the Infant Start, and the iBASIS-Video Interaction to Promote Positive Parenting [iBASIS-VIPP; Pickles et al. (11)], showed that symptomatic infants starting intervention before 15 months of age were significantly less likely to have a diagnosis of ASD at 3 years of age and showed more developmental gains in comparison to matched control groups of children who did not receive the intervention. These data are promising as they suggest that by providing intervention during the first phases of life, disability can be reduced and in a portion of individuals the full-blown diagnosis may perhaps even be prevented. However, it is important to note that for the current clinical model of ASD services, the identification of a risk is usually not sufficient, and a diagnosis is necessary to be able to access community intervention, thus only a limited number of children receive intervention within the developmental window most likely to significantly impact outcomes, that is during the first and second year of life.

Our report contributes to the literature on very early intervention, as it describes the clinical presentation and developmental outcomes of a 32-month-old-child who showed early signs of ASD during his first year of life and received a preemptive intervention based on an adapted version of the ESDM, the Infant Start (8), from 6 months of age. To our knowledge this is the first case study to report on an intervention starting so early in life and implemented in a community setting using an evidence-based intervention.

## Case description

Due to privacy issues we are not using the real name of the child. Here we will refer to the child as Francesco. Data related to Francesco’s medical history and developmental milestones were collected through clinical interviews, video-based observations, and standardized tools administered during clinical assessments at 6, 8, 14, 19, and 32 months.

Based on parent reports, from the first months of life, Francesco showed social and communicative atypicalities including diminished or almost absent eye contact, social orientation, and social smile as well as the presence of stereotyped motor behaviors and restricted interests. In his family there was no history of neurodevelopmental disorders or neurological conditions. The pregnancy proceeded regularly. Francesco was born at the 38th week of gestation without complications and an Apgar score of 8/9. Weight at birth was 3,140 g, length 48.5 cm, and head circumference 33.2 cm. Breastfeeding was suspended at 3 months due to hypogalease. Weaning was carried out successfully and the diet was varied. Sleep–wake rhythm was described as normal. According to parent report, social smiling appeared at about 4 months, but was generally scarce throughout the first year of life. As for posturomotor development, head control was achieved at about 4 months, sitting posture with support at 5½–6 months, complete rolling at 7 months, autonomous sitting posture at 8 months, quadrupedal movement at 9 months, autonomous walking at 14 months. In general, during the first year of life, spontaneous motility and motor initiative were slightly immature and asymmetrical as demonstrated by the prevalent use of the right side of the body. For example, during the 8-month assessment Francesco would reach for objects mainly with his right hand and roll only on his right side. In regard to the stages of language development, babbling emerged at 11 months after the child was specifically stimulated during intervention.

During the first months of life, Francesco’s parents consulted with several local professionals who attributed the mother’s concerns about her child’s social communication development to her own anxiety. Subsequently, Francesco’s parents consulted with one of the authors, an expert in early ASD identification and intervention when the child was 6 months of age. Francesco’s mother started worrying about his development when he was about 4 months old. She was concerned that Francesco seemed extremely passive. He would be found in his crib awake, without complaining nor trying to attract his parents’ attention. Francesco would spend time in the crib by flickering his hands in front of his face. His mother could not make eye contact with him, and only occasionally and partially would he respond to her social smile. Francesco did not orient his gaze toward his parents. He was annoyed when his parents tried to touch him, to pick him up or to caress him. He would get fractious when picked up from the front. He only tolerated being picked up when his back was held against his parents’ chest. Francesco was interested in visually inspecting cell phones, televisions or computers even when these devices were turned off. He used almost exclusively the right part of his body. For example, he grabbed objects only with his right hand. Plagiocephaly was present on the right side since birth. He rarely vocalized. His mother had consulted several pediatricians who had not noticed immaturity or atypicalities, but attributed the mother’s worries to anxiety. However, when two experts in early detection and treatment of ASD reviewed videos of interaction between Francesco and his mother, they confirmed his mother’s concerns. Indeed, even when Francesco was positioned in front of his mother, he did not orient toward her voice, nor did he establish eye contact with her, rather he remained with his head in a static position toward the left side, as if he had a stiff neck. He laughed without looking at his mother only after having been rocked by her. His mother confirmed that he usually laughed only after physical stimulation, without, however, directing his gaze toward the adult. This behavior is highly atypical, as children easily establish eye contact with the adult from birth and constantly respond to the adult’s smile by smiling back from the second month of life. After in presence and online observations, the above clinicians recommended further medical investigation and intervention. However, this time coincided with the beginning of the first COVID-19 Lockdown in Italy (March 2020). Therefore, it was not possible for the family to leave their home and access services. Thus, one of the authors started online parent mediated intervention based on Infant Start (8) and adaptation of the Early Start Denver Model [ESDM; Rogers and Dawson (10)].

The ESDM (10) is an evidence-based intervention validated in the USA [e.g., (12, 13)] and in several other countries around the world including Italy, where it has been implemented also in community settings [e.g., (14, 15)]. The ESDM is an empirically based intervention that fuses a relationship-focused developmental model with principles/practices of Applied Behavior Analysis. It uses a child-centered and responsive interactive style. It is delivered by adults within the context of play and daily routines in which highly precise naturalistic behavioral teaching is embedded, making this a Naturalistic Developmental Behavioral Intervention (NDBI), that is, one the most efficacious interventions for improving outcomes of children with ASD during the first phases of life (16). In Francesco’s case, the Infant Start (8), an adapted version of the ESDM for infants, was delivered. The program addressed the five elements of efficacious very early intervention described in Wallace and Rogers (17): (1) parent coaching, (2) frequency and length of intervention, (3) individualized, developmentally appropriate activities, (4) beginning the interventions as early as possible, and (5) increasing parental sensitivity and responsivity to infant cues. The Infant Start focuses on teaching parents strategies to improve atypicalities and immaturities identified in infants at risk for ASD including diminished eye contact, diminished communicative intent/communication, and unusual pattern of object exploration. Thus, Francesco’s parents were coached through five foundational intervention themes described in Rogers et al. (8) including: (1) joining into toy play to facilitate attention shifting from object to parent and parallel play, (2) encouraging flexible and varied actions and play to increase number and maturity of schemes used by the child, (3) increasing engagement and interaction to elicit communicative gesture, vocalizations, and integrated communicative behaviors for varied pragmatic intents, (4) developing the foundation of speech to increase the frequency of child vocalizations and shape specific consonant and vowel, (5) maximizing social attention to increase gaze, infant pleasure, and engagement in social interaction.

## Diagnostic assessment, details of the therapeutic intervention, follow-up and outcomes

**Figure 1:**
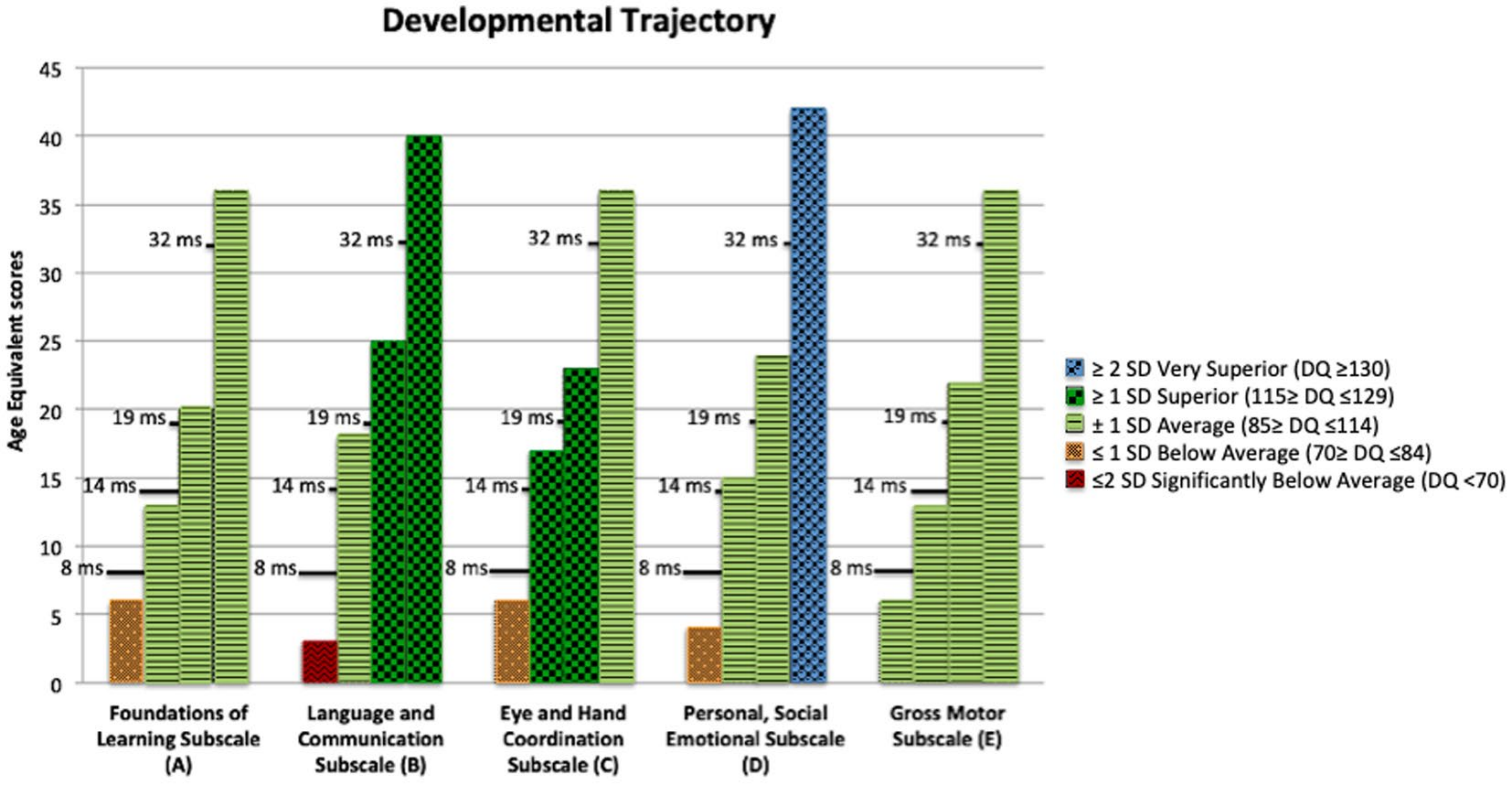
Griffiths III age equivalent (AE) scores at 8, 14, 19, 32 months of chronological age. The bars, representing the AE score at each evaluation, are colored differently in accordance with the standard deviation of the Developmental Quotient from the norm.

When Francesco was 8 months old, he was admitted to the Division of Child Neurology and Psychiatry of the Institute for Maternal and Child Health – IRCCS “Burlo Garofolo” in Trieste, Italy, a regional public institute for health care and scientific research. The evaluation was conducted by a child neuropsychiatrist expert in ASD and Neurodevelopmental Disorders in the early stages of life. The evaluation, which took place during a three-day hospitalization, included clinical observation, administration of standardized developmental tests [i.e., Griffiths Developmental Scales (18)], and instrumental neurological evaluation. During the evaluation Francesco took little interest in his parents and other adults. When the parents moved away from him, he showed no signs of awareness or dismay. Francesco entertained himself with stereotyped hand movements in front of his face, atypical rotations of his hands and repetitive behaviors with objects. For example, lying on the hospital bed, he repeatedly turned the cap of a bottle without varying his movements and without orienting himself toward the adults near him who were talking and trying to attract his attention. The Griffiths Developmental Scales (18) evidenced a highly immature level of development for Francesco’s age, corresponding to an equivalent age of development similar to a 4–5-month-old infant (see Figure 1). So, at 8 months Francesco was highly immature as regards his psychomotor and socio-communicative development. Atypicalities included poor eye contact, lack of response to social smile, poor awareness of the presence or absence of the adult, stereotyped hand movements and repetitive behaviors with objects, which could not be accounted for by sensory deficits such as blindness or deafness. Although Francesco was too young to undergo an ADOS-2 assessment (19), his behaviors met 4 out of 5 critical items on the SACS-R (20, 21), a research screening tool for young children with ASD (risk for ASD is hypothesized when 3 or more critical items are identified). In Francesco’s case the critical behaviors identified were: (1) atypical eye contact, (2) reduced reciprocal behaviors, (3) absent imitation behaviors, (4) reduced social smiling. The only critical item that was not met was failure to respond to name. As far as the instrumental tests are concerned, no asymmetries or epileptiform abnormalities were found in resting-state EEG. However, a slight immaturity in the organization of the bioelectric activity of the brain was found during sleep EEG. The MRI showed a mild plagiocephaly as well as a modest enlargement of the subarachnoid CSF spaces, especially in the frontal and vertex areas, in the base cisterns and ventricular cavities. At the audiometric evaluation, hearing appeared in the normal frequency range, and acoustic reflexes were present both ipsi- and contralaterally. The otoacoustic emissions were present bilaterally. At the eye examination visual acuity appeared normal and orthophoria was present for near objects. The child was able both to fix and follow a target. The anterior segment of the eye and the posterior pole were normal. However, an asymmetrical distribution of flash visual evoked potentials (VEPs) was found. Genetic testing for Fragile X Syndrome and SNP array analysis were negative. Francesco was discharged from the Hospital with a diagnostic risk for Autism Spectrum Disorder.

When Francesco was 9 months old, during the month of July, he underwent home intervention with one of the authors, 1–2 times a day for about 20–30 min at a time, based on Infant Start and ESDM strategies. The intervention was provided both directly (therapist – child) and mediated by the parents. During this month Francesco began to pay attention to the adult, to use eye contact both to request objects, to request the continuation of social games, and to share his interest with others. Pointing, used both to request and to share, also emerged. Francesco seemed involved and interested, but strongly passive, with little communicative initiative if not stimulated, and little motor initiative. Facial expressions were almost absent. Francesco showed a neutral gaze although he participated in the interaction with the other. It should be noted that Francesco communicated with his gaze and some gestures (e.g., pointing), but did not vocalize during the activities. He kept trying to put his lips together to produce vocalizations but could not produce sounds. This absence of vocalizations and babbling at 10–11 months of life denotes an important immaturity and is highly atypical. The therapist implemented vocalization stimulation strategies by standing in front of Francesco, while he was sitting in his high chair, and reproducing simple vocalizations for the child to imitate. These strategies seemed to have a positive effect on Francesco’s vocal development. However, Francesco continued to isolate himself when the adult was not intentionally stimulating him. He could remain in the cot or sitting in the stroller for a long time, even for 20–30 min looking into space without orienting himself toward or looking for other people near him.

In September, after the summer break, when Francesco was 11 months old, the family enrolled in services at the Intervention Centre of the IRCCS Stella Maris Foundation (Pisa). The intervention, based on the ESDM, was carried out by a team of professionals including a child neuropsychiatrist, two psychologists and a neuropsychomotricist (see Table 1 for the detailed scheme of his intervention program). The intervention continued to be supervised by the same therapist who had delivered the Infant Start since the child was 6 months of age. In the meantime, Francesco also started nursery school enrolling in a half day program, with the presence of a special education teacher for all the hours of his attendance. The collaboration with the school, through weekly supervisions conducted by one of the psychologists of the Centre, made it possible to coordinate the health professional intervention (IRCCS Stella Maris) and the educational program. Thus, it was possible to work in a coordinated way with common objectives so that Francesco practiced the skills acquired within 1:1 therapy also in the school context with educators and peers.

**Table 1:** Intervention program.

	1st year		2nd year	3nd year
	(6–8 ms)	(9 ms)	(11–20 ms)	(23–32 ms)
P-ESDM	2 h a week*		1 h a week	
ESDM		30 min twice a day	2 h a week	1 h a week
Nursery School			Half time	Full time
Supervision of School activities			1 h a week	1 h (until December)

Description of frequency of parent mediated intervention (P-ESDM), therapist delivered intervention (ESDM), and nursery school attendance across the years. *Sessions were delivered online.

Throughout the second year of life, Francesco underwent two global assessments, at the age of 14 and 19 months, which showed he had reached developmental skills within the typical range or above (see Table 2 for an overview of the Griffiths III Scales scores). It is not surprising that he improved in all areas of development, as the ESDM is a global intervention that addresses all developmental areas, including receptive and expressive communication, social interaction, imitation, play, cognition, motor skills, and personal independence. Moreover, at both assessments the ADOS-2 (19), considered “gold standard” for the detection of symptoms attributable to ASD, presented scores below threshold for the disorder with a low severity score index (see Table 2 for an overview of the ADOS-2 scores). However, clinically Francesco still presented some atypicalities including restricted interests, rigidity and occasionally, reduced reciprocity. Consequently, his intervention program was extended to the following year, but it included fewer hours of individual intervention and more involvement in regular school activities, which Francesco attended full time (see Table 1 for an overview of his intervention program).

**Table 2:** Developmental quotients (DQ), age equivalent (AE) scores and percentiles (%) for each of the Griffiths III subscales at 8, 14, 19, and 32 months of chronological age (CA).

Griffiths III		8 ms	14 ms	19 ms	32 ms
Foundations of learning subscale (A)	DQ	79	95	105	113
	AE	6	13	20	36
	%	8	37	63	79
Language and communication subscale (B)	DQ	68	112	120	123
	AE	3	18	25	40
	%	1	79	90	94
Eye and hand coordination subscale (C)	DQ	82	115	125	111
	AE	6	17	23	36
	%	10	84	95	75
Personal, social emotional subscale (D)	DQ	76	104	113	136
	AE	4	15	24	42
	%	5	61	79	>99
Gross motor subscale (E)	DQ	86	90	114	110
	AE	6	13	22	36
	%	16	25	81	75
General quotient	GQ	74	95	117	123
	AE	5	13	23	36
	%	4	37	87	94
ADOS-2					
Module			Toddler	Toddler	Module 2
Social affect			2	4	1
Restricted repetitive behaviors			4	2	1
Total score			6	6	2
Calibrated severity score			3	3	1
VABS-II					
Communication domain			82	108	106
Daily living skills domain			80	99	90
Socialization domain			80	89	101
Motor skills domain			85	92	98
Adaptive behavior composite			79	96	111

Toward the end of the school year, at the age of 32 months, Francesco underwent an additional developmental and diagnostic assessment. The ADOS-2 confirmed scores under the cut-off for ASD (see Table 2). It is worth noting that at this time point, Francesco was able to sustain an ADOS-2 module 2, which is used for children with phrase speech abilities. Indeed, at the assessment with the Griffiths Scales, Francesco performed on the Language and Communication subscale as a child with an equivalent age of 40 months [Developmental Quotient (DQ) 123]. Francesco was able to communicate using complex sentences with advanced grammar markers, such as verbs correctly conjugated in the present, past and future tenses, as well as combining complex sentences using grammar connectors such as “why,” “when,” and “but.” He was able to initiate a conversation or respond taking advantage of the interlocutor’s comments by adding content that allowed the other person to expand the exchange, therefore showing an optimal level of reciprocity. He was also able to integrate verbal language with non-verbal communication methods such as eye contact and gestures. The use of gestures appeared particularly advanced too. Francesco could use and understand both conventional and descriptive gestures. His skills were outstanding also at the Personal, Social Emotional Subscale of the Griffiths (Equivalent age of 42 months; DQ 136), where he showed that he was able to assume the perspective of the other person, and to understand some moral principles (e.g., stealing is bad, helping is good). Francesco not only caught up with his peers but also placed himself on the higher percentiles in most areas of development (see Table 2 on the Griffiths III scores). Indeed, he obtained a general development quotient of 123 (the norm is between 85 and 115) with an equivalent age of 36 months, placing his skills at the 94th percentile (i.e., his developmental abilities were higher than 94% of the children his age). In Figure 1, it is possible to observe how toward the end of his first year of life and in his second year of life immaturities decreased. At first Francesco reached an adequate level for his age (14-month assessment) and subsequently (19 and 32-month assessments) he reached above average linguistic and cognitive abilities. It is noteworthy that not only was his performance during the Griffiths assessment good, but also his adaptive functioning, as reported by parents at the Vineland Adaptive Behavior Scales – 2nd edition Survey Interview Form (22), was adequate for his chronological age (Adaptive Behavior Composite Score: 111), meaning that he was able to generalize the abilities learnt during the intervention and use them also in everyday life.

## Conclusion

In this report we described the case of an infant showing early behavioral signs of ASD, who received preemptive intervention from the age of 6 months. Francesco’s parents were concerned because his developmental profile not only presented immaturities but also a significant deviation from a typical developmental trajectory. Many of his behaviors were in fact atypical, including intolerance to touch, prolonged visual interests in inanimate objects such as cell phones and TVs, stereotyped hand movements, repetitive behaviors with objects, scarce interest in people including parents, absent or limited use of eye contact to request or share, reduced modulation of facial expressions, and rigidity in maintaining certain body postures. In order to address these concerns Francesco received a parent mediated low intensity intervention based on the Infant Start and the ESDM, in combination with state-funded educational services. Developmental changes were documented longitudinally on behavioral measures including the ADOS-2, the Griffiths-III, and the Vineland Adaptive Behavior Scales at 8, 14, 19, and 32 months by different teams of professionals who were not involved in the intervention program. Based on these measures and clinical judgment, Francesco showed a significant improvement in developmental skills and did not meet criteria for a diagnosis of ASD when formally evaluated at 32 months of age. Although these results cannot be generalized, the perspective of diminishing disability and preventing a diagnosis is in line with previous accounts (8, 9). In this respect, Rogers’s et al. (8) pilot study on Infant Start, showed that at 36 months the treated group reported higher gains in DQs and much lower rates of ASD than a similarly symptomatic group who did not enroll in the treatment study at 9 months. Similarly, Whitehouse et al. (9) showed that participation in a preemptive intervention for symptomatic infants starting at 9 months of age led to reduced ASD symptom severity and lowered the odds of an ASD diagnosis at 3 years of age.

Considering the low intensity of specialized services involved in Francesco’s treatment and the absence of side effects, in combination with the positive results shown in previous group studies (8, 9), a wider application of such an approach at the community level could be a feasible practice. The current clinical model, which requires a diagnosis before accessing intervention often leads to beginning treatment after the second year of life, when brain plasticity is not at its maximum capacity anymore. Our case report, in line with current research, supports the implementation of universal screening during the first year of life (23, 24) and the use of preemptive intervention to promote optimal outcomes for infants with early social communication atypicalities. Thus, a controlled trial is a fundamental next step for demonstrating the feasibility and efficacy of this model in Italian health services. Not only does preemptive intervention have the potential to improve childhood and adulthood long-term outcomes, but it could also reduce the costs of lifelong services.

## Data availability statement

The raw data supporting the conclusions of this article will be made available by the authors, without undue reservation.

## Ethics statement

Ethical review and approval was not required for the study on human participants in accordance with the local legislation and institutional requirements. Written informed consent to participate in this study was provided by the participants’ legal guardian/next of kin. Written informed consent was obtained from the individual(s), and minor(s)’ legal guardian/next of kin, for the publication of any potentially identifiable images or data included in this article.

## Author contributions

CC and NC drafted the manuscript. NC, AC, VC, RD, and MP assessed Francesco at different time points. CC and CP treated the patient and worked with the family. FL offered consultation during the administration of the intervention. CC contacted the family for approval and coordinated the approval of the final draft. All authors contributed to the article and approved the submitted version.

## Funding

This work has been partially supported by Grant RC, Italian Ministry of Health.

## Conflict of interest

The authors declare that the research was conducted in the absence of any commercial or financial relationships that could be construed as a potential conflict of interest.

## Publisher’s note

All claims expressed in this article are solely those of the authors and do not necessarily represent those of their affiliated organizations, or those of the publisher, the editors and the reviewers. Any product that may be evaluated in this article, or claim that may be made by its manufacturer, is not guaranteed or endorsed by the publisher.
